# *TPMT* and *NUDT15* testing for thiopurine therapy: A major tertiary hospital experience and lessons learned

**DOI:** 10.3389/fphar.2022.837164

**Published:** 2022-09-23

**Authors:** Liuh Ling Goh, Chia Wei Lim, Khai Pang Leong, Kiat Hoe Ong

**Affiliations:** ^1^ Molecular Diagnostic Laboratory, Personalized Medicine Service, Tan Tock Seng Hospital, Singapore, Singapore; ^2^ Department of Rheumatology, Allergy & Immunology, Tan Tock Seng Hospital, Singapore, Singapore; ^3^ Department of Haematology, Tan Tock Seng Hospital, Singapore, Singapore

**Keywords:** TPMT, NUDT15, thiopurine, pharmacogenetics, precision medicine

## Abstract

Variants in thiopurine methyltransferase (*TPMT*) and nudix hydrolase 15 (*NUDT15*) are associated with an accumulation of cytotoxic metabolites leading to increased risk of drug-related toxicity with standard doses of thiopurine drugs. We established *TPMT* and *NUDT15* genetic testing for clinical use and evaluated the utilization, service outcomes and potential value of multi-gene PGx testing for 210 patients that underwent pharmacogenetics (PGx) testing for thiopurine therapy with the aim to optimize service delivery for future prescribing. The test was most commonly ordered for Gastroenterology (40.0%) and Neurology (31.4%), with an average turnaround time of 2 days. Following testing, 24.3% patients were identified as intermediate or poor metabolizers, resulting in 51 recommendations for a drug or dose change in thiopurine therapy, which were implemented in 28 (54.9%) patients. In the remaining patients, 14 were not adjusted and 9 had no data available. Focusing on drug gene interactions available for testing in our laboratory, multi-gene PGx results would present opportunities for treatment optimization for at least 33.8% of these patients who were on 2 or more concurrent medications with actionable PGx guidance. However, the use of PGx panel testing in clinical practice will require the development of guidelines and education as revealed by a survey with the test providers. The evaluation demonstrated successful implementation of single gene PGx testing and this experience guides the transition to a pre-emptive multi-gene testing approach that provides the opportunity to improve clinical care.

## Introduction

Pharmacogenetics (PGx) testing can aid healthcare providers in selecting appropriate treatment and dosing (Johnson, 2003). The genotyping methods can adopt a reactive or pre-emptive testing approach. In the reactive testing model, genotypes for specific variant(s) related to the drug being started at that time are measured. On the other hand, genotypes for multiple pre-specified variants related to several medications are measured simultaneously in the pre-emptive testing model (Dunnenberger et al., 2015). The Personalized Medicine Service (PMS), established in 2015 in our institution to enable personalized treatment to each patient, initiated the PGx testing service to guided pharmacotherapy for routine practice. The reactive testing approach was adopted for the service due to issues related to the incorporation of clinical decision support and PGx results in the electronic medical record (EMR). One of the drug gene interactions implemented is thiopurine S‐methyltransferase (*TPMT*) and nucleoside diphosphate-linked moiety X-type motif 15 (*NUDT15*) for thiopurines.

Thiopurine drugs (azathioprine, mercaptopurine, and thioguanine) are used as chemotherapeutic agents for treatment of certain types of malignancies (e.g., acute lymphoblastic leukaemia) and also as immunosuppressors in autoimmune disorders including inflammatory bowel disease (IBD), such as Crohn’s disease and ulcerative colitis, and psoriasis (Karran and Attard, 2008; Abaji and Krajinovic, 2017; de Boer et al., 2018). Additionally, thiopurine drugs are used in organ transplant recipients to help prevent the immune system from attacking the transplanted organ. Despite their efficacy, a potential complication of treatment with thiopurine drugs is adverse drug reactions, namely hematopoietic toxicity and the possibility of severe or life‐threatening myelosuppression (Weinshilboum and Sladek, 1980; Goldberg and Irving, 2015). Some of these adverse reactions are known to be caused by individual differences in thiopurine metabolism, which is related to genetic polymorphisms of the enzymes (Dervieux et al., 1999; Dubinsky et al., 2000).

Thiopurines are pro-drugs that require extensive metabolism to form thioguanine nucleotides which are incorporated into DNA to exert their cytotoxic action. The TPMT enzyme functions mainly to inactivate these drugs; thus, a deficiency or low activity of TPMT results in elevated levels of the thiopurine active metabolites with resultant toxicities (Coulthard and Hogarth, 2005; Karran and Attard, 2008). The gene encoding the TPMT enzyme is affected by germline polymorphisms that lead to varied levels of enzyme activity among individuals. More than 40 different *TPMT* alleles (TPMT*2-*41) have been reported in individuals with TPMT deficiency (Spire-Vayron de la Moureyre et al., 1998; Iu et al., 2017). Most of these variants are associated with decreased TPMT activity, relative to the wild-type allele (TPMT*1). Two particular alleles, TPMT*3A and TPMT*3C, underlie more than 90 percent of cases of the condition (Meng et al., 2018). Nearly all patients with two inactive *TPMT* alleles experience severe or life‐threatening myelosuppression with standard thiopurine doses. Patients with one inactive *TPMT* allele have higher levels of thioguanine nucleotide metabolites and increased risk of myelosuppression as compared with patients who are homozygous for wild-type *TPMT* alleles (Relling et al., 1999; Evans et al., 2001; Ujiie et al., 2008). The Clinical Pharmacogenetics Implementation Consortium (CPIC) provides therapeutic recommendations to guide thiopurine dosing based on *TPMT* genotypes (Relling et al., 2013).

The frequencies of *TPMT* genetic polymorphisms vary in different ethnic groups and TPMT deficiency cannot explain the higher incidence of adverse reactions in East Asian patients (Collie-Duguid et al., 1999; Colombel et al., 2000; Takatsu et al., 2009; Booth et al., 2011). NUDT15 deficiency has emerged as another important determinant of thiopurine intolerance, most frequently in those of Asian and Hispanic descent (Yang et al., 2014; Yang et al., 2015). It negatively regulates thiopurine activation, with loss-of-function *NUDT15* variants leading to accumulation of active metabolites and increased cytotoxicity (Moriyama et al., 2016; Valerie et al., 2016; Man et al., 2019). The NUDT15*3 deficient allele was the first variant to be identified that is strongly associated with thiopurine-toxicity (Yang et al., 2014). This association was affirmed by multiple independent studies (Tanaka et al., 2015; Kakuta et al., 2016; Zhu et al., 2016; Kakuta et al., 2018; Zhang et al., 2018). Furthermore, patients who were homozygous for NUDT15*3 had nearly perfect sensitivity and specificity for severe alopecia (Kakuta et al., 2016). Based on a growing body of evidence, CPIC has recently updated the existing thiopurines prescribing guideline based on both *TPMT* and *NUDT15* genotypes (Relling et al., 2019).

While *TPMT* gene testing was routinely ordered to assess TPMT enzyme function for thiopurine therapy, *NUDT15* testing and interpretation was not available and provided the opportunity to develop an in-house assay customized to the local needs of patients and providers. As part of the PMS program, the Molecular Diagnostic Laboratory developed clinical grade testing for *TPMT* and *NUDT15*, and established a standardized process for test ordering and returning of results in the EMR. In this article, we described our experience with the clinical implementation of *TPMT* and *NUDT15* testing in our institution. In particular, we aimed to assess the clinical impact of the test by evaluating the frequency of clinically relevant findings that resulted in subsequent management recommendations and changes in pharmacotherapy. Additionally, we characterized the potential utility and relevance of multi-gene PGx test among these patients who undergone *TPMT* and *NUDT15* testing by examining their medication records. Finally we sought to identify enablers of PGx implementation through a questionnaire survey from our test users.

## Materials and methods

### Study population and data collection

This study is a retrospective review of 210 patients who underwent *TPMT* and *NUDT15* genotyping at Tan Tock Seng Hospital (TTSH) from 2^nd^ November 2016 to 28^th^ December 2018. Data collected from the EMR included patients’ demographics, diagnosis, current medications and discontinued medications, PGx test results and discipline of ordering physician. Performance indicators such as service utilization and turnaround time (TAT) were collected from the laboratory information system from 2 November 2016 to 31 December 2020. This study was approved and deemed exempt for patient consent by the Institutional Ethics Committee of TTSH.

### *TPMT* and *NUDT15* testing

Genotyping was performed in-house as a clinical test for standard of care at Molecular Diagnostic Laboratory, which is accredited by the College of American Pathologists (CAP). Genomic DNA was extracted using QIAamp DNA Mini QIAcube Kit (Qiagen, Germany). The laboratory-developed *TPMT* (NM_000367.3) and *NUDT15* (NM_018283.3) genotyping panel was designed to test for TPMT*3B c.460G>A (rs1800460), TPMT*3C c.719A>G (rs1142345) and NUDT15*3 c.415C>T (rs116855232) single nucleotide polymorphisms (SNPs) using TaqMan-based real-time PCR (Thermo Fisher Scientific, United States). Amplification was performed in a 10 μl reaction volume containing 5 μl of 2X TaqMan Genotyping Master mix, 0.5 μl 20X TaqMan SNP assay, 2.5 μl nuclease-free water and 2 μl of DNA (5 ng/μl). Thermal cycling was performed with denaturation at 95°C for 10 min, followed by 50 cycles of 95°C for 15 s and 60°C for 90 s. Genotype calling and data analysis were performed using TaqMan Genotyper software. The genotype data were translated to star alleles and phenotypes in alignment with guidelines at Pharmacogenomics Knowledgebase (PharmGKB) website (https://www.pharmgkb.org). Recommended change in prescribing (i.e., dose or drug selection) was adapted from clinical guidelines published by the CPIC and the Royal Dutch Association for the Advancement of Pharmacy—Pharmacogenetics Working Group (DPWG) (Table 1) (Relling et al., 2013; Relling et al., 2019). Results were returned in standard report formats in the EMR that included patient genotype, metabolizer phenotype and clinical recommendation.

**TABLE 1:** Recommendations based on CPIC and DPWG guidelines.

Phenotype	TPMT Normal metabolizer (NM)	TPMT Intermediate metabolizer (IM)	TPMT Poor metabolizer (PM)
NUDT15 Normal metabolizer (NM)	Treat with label recommended initial dose of a thiopurine drug	Treat with a lower initial dose. Subsequent doses should be adjusted based on the degree of myelosuppression and disease-specific guidelines	Treat with drastically reduced initial dose for malignant conditions. Subsequent doses should be adjusted based on the degree of myelosuppression and disease-specific guidelines. For non-malignant conditions, alternative non-thiopurine immunosuppressant therapy is recommended
NUDT15 Intermediate metabolizer (IM)	Treat with a lower initial dose. Subsequent doses should be adjusted based on the degree of myelosuppression and disease-specific guidelines	Treat with a lower initial dose. Subsequent doses should be adjusted based on the degree of myelosuppression and disease-specific guidelines	Treat with drastically reduced initial dose for malignant conditions. Subsequent doses should be adjusted based on the degree of myelosuppression and disease-specific guidelines. For non-malignant conditions, alternative non-thiopurine immunosuppressant therapy is recommended
NUDT15 Poor Metabolizer (PM)	Treat with drastically reduced initial dose for malignant conditions. Subsequent doses should be adjusted based on the degree of myelosuppression and disease-specific guidelines. For non-malignant conditions, alternative non-thiopurine immunosuppressant therapy is recommended	Treat with drastically reduced initial dose for malignant conditions. Subsequent doses should be adjusted based on the degree of myelosuppression and disease-specific guidelines. For non-malignant conditions, alternative non-thiopurine immunosuppressant therapy is recommended	Treat with drastically reduced initial dose for malignant conditions. Subsequent doses should be adjusted based on the degree of myelosuppression and disease-specific guidelines. For non-malignant conditions, alternative non-thiopurine immunosuppressant therapy is recommended

CPIC, clinical pharmacogenetics implementation consortium; DPWG, Royal Dutch Association for the Advancement of Pharmacy—Pharmacogenetics Working Group; NM, normal metabolizer; IM, intermediate metabolizer; PM, poor metabolizer.

### Outcome measures and data analysis

A cross sectional analysis was performed using data extracted from EMR and laboratory results database. Data extracted were de-identified for analysis. The proportion of patients with actionable PGx variants and PGx-based management recommendations were measured. Genotype and phenotype frequencies were compared to data from CPIC guideline supplements and PharmGKB. For NUDT15*3, the prevalence was also compared to data from 506 healthy individuals (201 Chinese, 179 Indians and 126 Malays) from TTSH biobank. The effectiveness of implementation was measured by tracking the number of revised prescriptions based upon genotype results. Information on prescription were retrieved from the EMR. PGx-based recommendations were considered implemented with a prescription change within 3 months of genotyping. Other implementation metrics tracked include service indicators such as test utilization, TAT and provider satisfaction. Ninety-eight ordering physicians were invited to complete a questionnaire to assess their satisfaction with the service, and to identify enablers to service implementation.

The potential value of PGx multi-gene testing was determined by the proportion of medications with PGx guidance being used in this cohort. Data from the medication records within 3 months of genotyping was used. We restricted to medications with CPIC level 1A guidelines, and with PGx variants for 10 genes that can be tested in-house (Supplementary Table S1). Using patient-level data, we quantified the proportion of patients who had orders of 0, 1, 2, 3, 4 or more medications with actionable PGx guidance. We then calculated the percentage of drugs being prescribed in this cohort.

## Results

### Implementation process and patient characteristics

To prepare for the service, *TPMT* and *NUDT15* genetic testing was developed and validated according to CAP guidelines. A standardized institutional process for test ordering, testing and reporting was established. The test was orderable in the institution’s electronic order entry system and was not reimbursed (approximately SGD$128 out of patient’s pocket). An official email on the new test with relevant publications was sent to all clinicians and pharmacists in our institution. Test information and laboratory phone number were made available in our intranet website to ordering physicians. Laboratory personnel were trained to respond to enquiries related to testing and results interpretation. Education talks were organised to increase awareness and promote the adoption of PGx-guided therapy. We report the results of a retrospective analysis of 210 patients that underwent *TPMT* and *NUDT15* testing in TTSH from November 2016 to December 2018. Patients were adults who received clinical PGx testing as standard of care. There was no formal inclusion/exclusion criteria. Once ordered in the electronic order system, blood sample was sent to the laboratory for processing and analysis. Reports were prepared, reviewed and signed off by the laboratory director. Genotype and phenotype results were available as discrete data in the EMR (Supplementary Figure S1). Clinical decision support was not built within the EMR due to infrastructure limitations for building computational algorithms and is limited.

Singapore has a diverse population comprising three major ethnic groups: Chinese (East Asian), Malay (Southeast Asian) and Indian (South Asian). The demographics of our population differ from many institutions abroad. This study included 104 males and 106 females with a median age of 52 years, ranging from 18 to 83 years, and over half were Chinese (67.6%, N = 142/210). Patient characteristics are presented in Table 2. The majority of the patients were diagnosed with non-malignant conditions (>90%), such as IBD and myasthenia gravis.

**TABLE 2:** Demographic information of patients.

Total	N = 210 (100%)
Age, years	
Median	52
Range	18–83
Gender	
Female	106 (50.5%)
Male	104 (49.5%)
Race	
Chinese	142 (67.6%)
Indian	33 (15.7%)
Malay	25 (11.9%)
Others^#^	10 (4.8%)
Diagnosis	
Inflammatory bowel disease	62 (29.5%)
Myasthenia gravis	34 (16.2%)
Autoimmune hepatitis/ Jaundice/ Pancreatitis	12 (5.7%)
Leukemia	12 (5.7%)
Rheumatic diseases	9 (4.3%)
Myelitis	8 (3.8%)
Dermatologic conditions	7 (3.3%)
Others	38 (18.1%)
Not available	28 (13.3%)

# Refers to minorities in the Singapore population.

**FIGURE 1:**
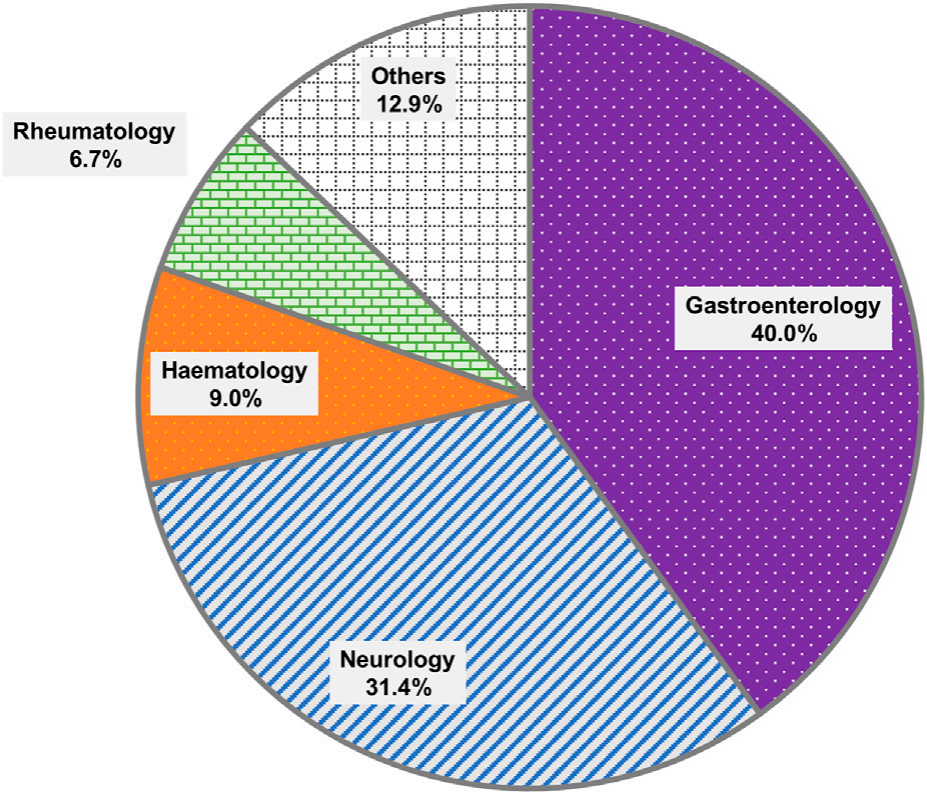
Distribution of test orders by clinical specialties.

The distribution of orders by clinical specialty was presented in Figure 1. The most common specialties requesting testing were Gastroenterology (N = 84) and Neurology (N = 66), collectively accounting for 71.4% of all orders. This was likely due to the greater number of patients seen for the treatment of IBD than leukemia. The next most common specialties included Haematology (N = 19) and Rheumatology (N = 14).

### Testing results

**FIGURE 2:**
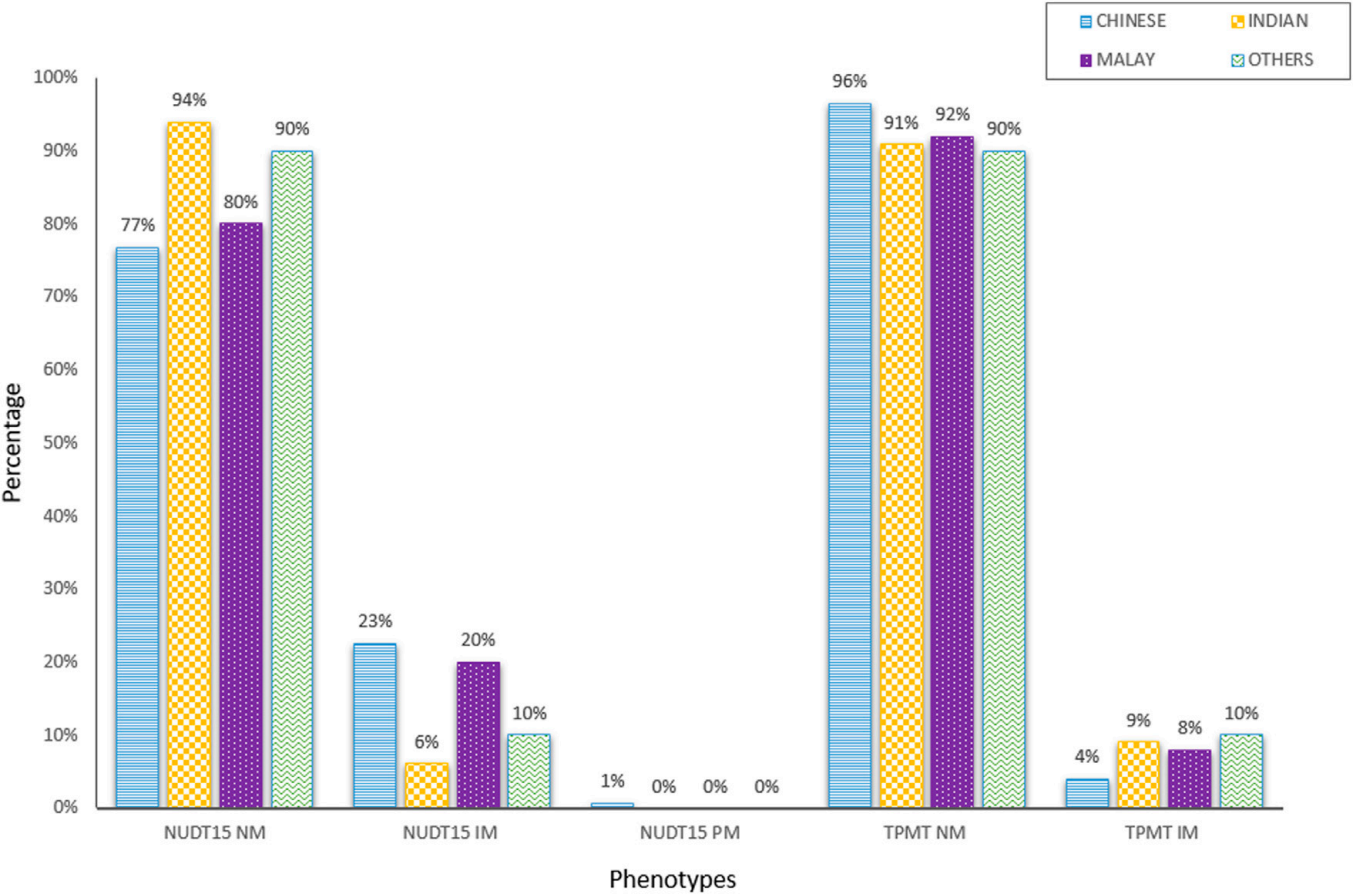
Distribution of NUDT15 and TPMT diplotypes and phenotypes among different ethnic groups (N = 210).

This study aims to measure the proportion of patients with actionable results, which were defined as phenotypes with a recommended change in prescription. Figure 2 showed the distribution of diplotypes and phenotypes among Chinese, Indian and Malay subjects. The NUDT15*3 allele was more prevalent and accounted for approximately 19.5% of intermediate and poor metabolizers (N = 41) in this cohort. Overall, the allele frequencies were similar to those reported in the Exome Aggregation Consortium (ExAC) database and were in concordance with the *NUDT15* genotypes evaluated in a local cohort of 506 healthy individuals from our biobank with 18.3% of intermediate and poor metabolizers (Supplementary Figure S2). The prevalence of TPMT*3 was significantly lower than that of *NUDT15*, which accounted for approximately 5.2% (N = 11) of intermediate metabolizers. None were poor metabolizers. However, Indians were noted to have a higher prevalence of TPMT*3 (9.09%, N = 3/33) than NUDT15*3 (6.06%, N = 2/33). Among those actionable genotypes, only one patient was tested positive for both *NUDT15* (*1/*3) and *TPMT* (*1/*3C). By having both *TPMT* and *NUDT15* in the test panel, the positivity rate for the prediction of thiopurine toxicity was increased to 24.3% (N = 51/210). This denotes the proportion of patients where pharmacogenetic testing for both *TPMT* and *NUDT15* variants had clinically relevant management implications in a clinical practice setting. Compared to only testing *TPMT*, this represents an increase in the diagnostic yield by almost 19%.

### Clinical outcomes

It was of interests to take the perspective of clinician making a prescribing decision for patient with actionable results. Following testing, 51 patients predicted to have intermediate or poor TPMT/NUDT15 activities were recommended to have their dose adjusted or select alternative drug. For these patients, we evaluated prescription change within 3 months of genotyping to gauge the recommendation acceptance rate. Within this time frame, data was not available for 17.6% (N = 9) of the patients. Prescription change was observed in 54.9% (N = 28) of the patients and 27.5% (N = 14) of them were not adjusted (not shown). This observation did not account for clinical factors outside of the drug and genotype. Other factors such as clinical indication and drug interactions may have a significant role in how or whether PGx results were applied. During this period, there were no notifications from clinicians on clinical or psychological harms resulting from the testing.

**FIGURE 3:**
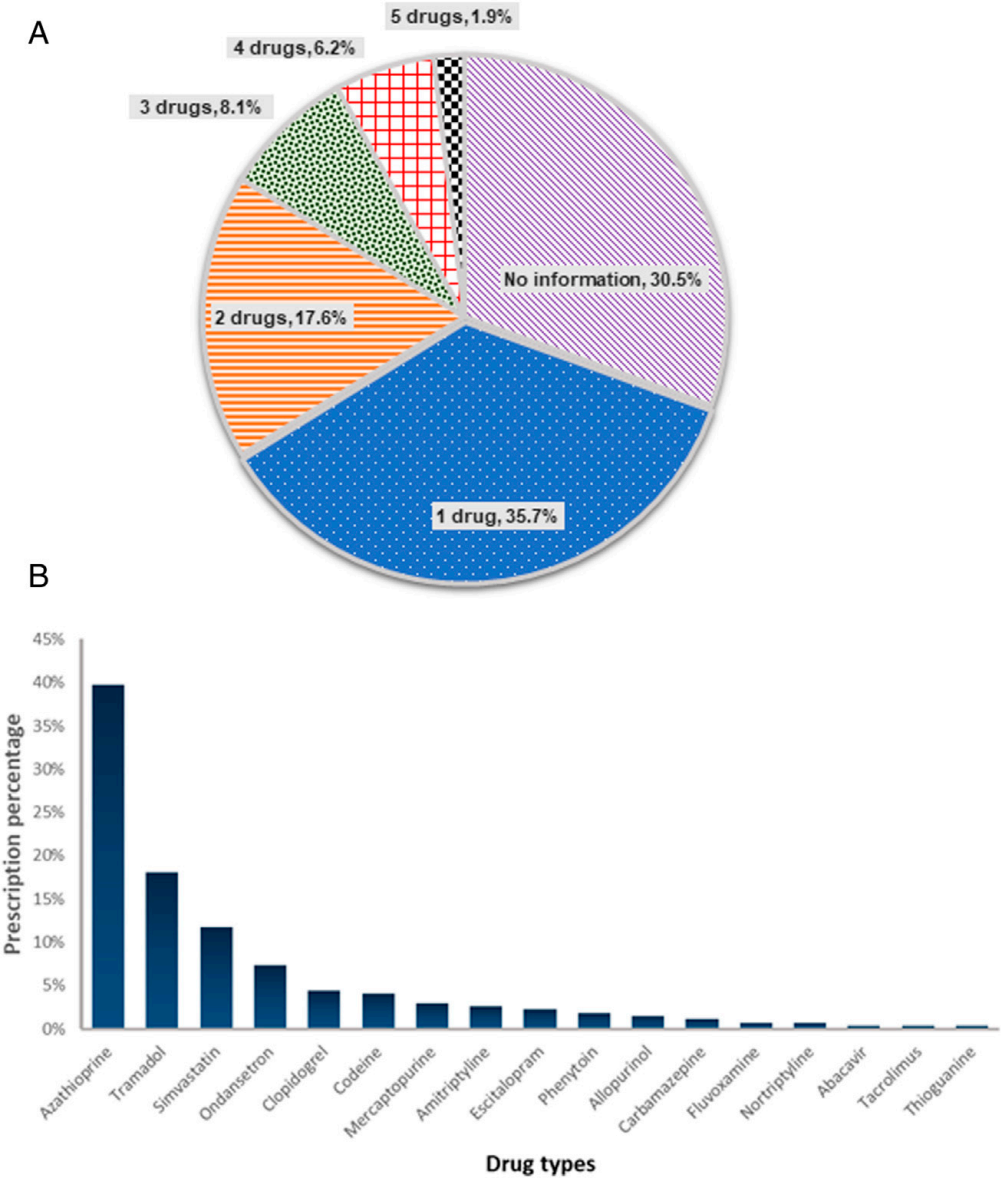
Potential of PGx multi-gene testing **(A)** Number of concurrent medications with PGx guidance being ordered per patient (N = 210) **(B)** Prescription pattern of actionable drugs in this patient cohort (N = 210). Actionable drugs refer to drugs with CPIC level 1A guidelines, and can be tested in-house.

Multi-gene assays provide more PGx data than single gene tests at similar cost. To characterize the potential value of PGx multi-gene testing, we examined the medication orders in this cohort, focusing on medication with CPIC guideline or FDA recommendations. We also confined the medication list with gene targets that were available for testing in our laboratory (*CYP2C19, CYP2C9, CYP2D6, CYP3A5, HLA-A, HLA-B, NUDT15, SLC O 1B1* and *TPMT*) (Supplementary Table S1). Of the 17 medications that met the criteria, a total of 272 prescriptions were made. The vast majority (69.5%, N = 146) of patients had at least one order for a drug with actionable PGx guidance, and 71 patients (33.8%) had 2 or more actionable medications (Figure 3A). We next evaluated the prescription pattern of drugs with PGx guidance in this cohort. Excluding thiopurines, the medications most often prescribed were tramadol, simvastatin and ondansetron (Figure 3B). About 30.1% (N = 84/272) of the prescriptions (tramadol, ondansetron, codeine, fluvoxamine and nortriptyline) involved drugs metabolized by CYP2D6. This was followed by 6.6% (N = 18/272) of clopidogrel and escitalopram metabolized by CYP2C19. Amitriptyline was affected by CYP2D6 and CYP2C19 enzymes (N = 7). Overall, CYP2D6 and CYP2C19 enzymes metabolized 40.1% (N = 109/272) of medications prescribed in this cohort.

### Service performance indicators

**FIGURE 4:**
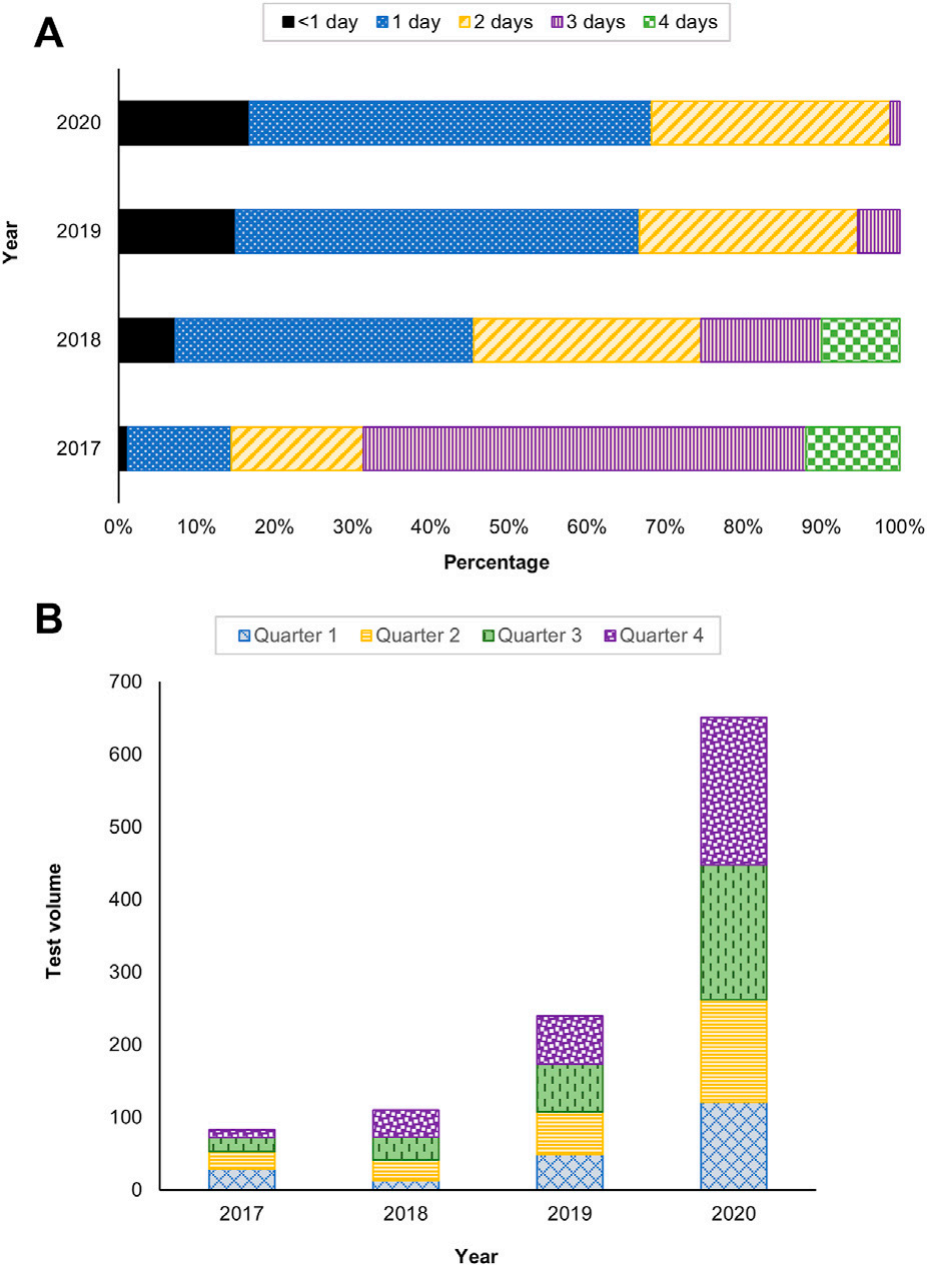
Service performance indicators **(A)** Turnaround time (TAT) and **(B)** Test utilization between 2017 and 2020.

The TAT, test utilization and provider satisfaction were evaluated as performance indicators of the service. The TAT is defined as the time from specimen receipt to reporting. In 2017, 31% of the tests had a TAT within 2 days and had improved over the years to 99% in 2020 (Figure 4A). An increase in uptake of the testing was also observed, with 2.2 and 2.7 fold increase in 2019 and 2020, respectively (Figure 4B).

A survey with ordering clinicians was conducted to assess service satisfaction (Table 3). In total, 14 out of 98 providers responded. Satisfaction ratings were high with all the participants agreeing that the PGx information provided in the report was appropriate and useful. Among these, 71% indicated that they followed the recommendations reported. All the participants agreed that the use of PGx has an impact on the management of their patients and expected the usage to increase in their specialties. The majority indicated that the development of protocols and guidelines (93%) will improve their understanding of PGx in clinical practice. Clearer guidelines will also increase the use of PGx in their specialty (79%). Many expressed interest to participate in educational PGx workshop organized in-house (86%).

**TABLE 3:** Perceived clinical utility of Pharmacogenetics (PGx) testing and implementation (N = 14).

Questions on clinical PGx testing	Response (%)
Do you consider the provided PGx information appropriate and useful?	Yes (100%)
With regards to the PGx test results and recommendations	1. Follow the indications reported (71%)
	2. Do not follow the exact indications but consider the genetic result (29%)
Do you think that the use of PGx has an impact on the management of your patient?	Yes (100%)
Do you think that in the following years the use of PGx will increase in your specialty?	Yes (100%)
Which of the following will improve your understanding about the utility of PGx in clinical practice?	1. Development of protocols and guidelines (93%)
	2. Education, sessions or specific courses (86%)
	3. Contact with Personalized Medicine Services (57%)
	4. Development of research projects related to PGx (50%)
Which aspects do you think could increase the use of PGx in your specialty?	1. Clearer guidelines about the use of PGx (79%)
	2. Greater level of evidence about its clinical validity and utility (71%)
	3. Evaluation of the cost-effectiveness of the use of PGx (64%)
	4. Subsidy or lower cost of the test (57%)
	5. Time of response (50%)
If an educational PGx workshop is organized in-house, would you be keen to participate?	Yes (86%)

## Discussion

Service for clinical testing of *TPMT* and *NUDT15* genotypes for thiopurine therapy was made available to physicians and their patients in our institution. The study identified 24.3% of patients that underwent testing had actionable results, with more 50% of them having a prescription change within 3 months of testing. Appropriate indicators were monitored to ensure service stability and efficiency prior to expanding the current PGx service. The majority of tests were ordered by non-haematology/oncology providers. There was more than 2 fold increase in test volume in recent years. Cost is often cited as a barrier to implementation. Although the testing was ‘out of pocket’ for the patients, we have seen a gradual increase in test ordering over the years, likely due to increased awareness and clinical utility. Based on our experience with other tests that are reimbursed or subsidized, the removal of cost to the patients will further increase the uptake. The increased test utilization contributed to the shortened TAT as the test can be performed sooner in batches.

The success of the implementation was firstly attributed to the choice of a good model for implementation in our setting. Drug gene pair with clinical validity and allele frequency of the tested SNPs in local population are imperative to clinical adoption of the service. In agreement with published data, risk alleles in *NUDT15* explain the majority of thiopurine-related myelosuppression in Asian (Moriyama et al., 2016). For the choice of variants, NUDT15*3 is a robust and obvious candidate for clinical application as all the reports to date indicate that thiopurine-induced leukopenia and severe alopecia are inevitable in patients homozygous for this variant. Moreover, this variant is prevalent in East Asian and hence relevant for the demographics of our population (Yang et al., 2014; Kakuta et al., 2018; Zhang et al., 2018). Recently, three additional variants, namely, NUDT15 p. Arg34Thr, p. Lys35Glu, and p. Gly17_Val18del, were observed in five children with acute lymphocytic leukaemia in Singapore, Taiwan, and the United States (Moriyama et al., 2016). These variants are not common (<1%) but can be rapidly added into our panel with sufficient evidence of clinical relevance. Although *NUDT15* was not included in the CPIC guidelines when we implemented the service, the early engagement of stake holders and its relevance in our population were critical to the success of our service.

Establishing a clinical grade test in-house with a clear workflow for ordering and integrating the results into the EMR is vital to the success of the implementation. One issue associated with outsourcing the test is the return of results in portable document format which is difficult to integrate the data as discrete field in the EMR. Although the current infrastructure does not allow building of CDS within our EMR, having the results in the system as discrete data was found to be reasonably effective for clinicians to retrieve the results. For test development in-house, the laboratory had to decide on the selection of the testing platform, variants to be tested as well as how to effectively report the results and interpretations. The genotyping approach was adopted in view of its simplicity of analysis, short TAT, lower costs and adaptability. The development of in-house testing builds local expertise and accumulates the knowhow that will be invaluable in future expansion of the service.

The addition of *NUDT15* genotyping to *TPMT* likely contributed toward further reducing the incidence of potentially lethal adverse drug reactions. The loss of function NUDT15*3 accounted for approximately 19.5% of intermediate and poor metabolizers in our population, which is in line with previous reports (Zhu et al., 2016; Kakuta et al., 2018; Zhang et al., 2018). Intriguingly, Indians were noted to have a higher prevalence of TPMT*3 (9.09%) than NUDT15*3 (6.06%). This observation differed from the data reported in the ExAC database where South Asian has a higher allele frequency in NUDT15*3 than TPMT*3. Having both genes in the panel identified variants relevant for thiopurine therapy in 24.3% of tested patients. This represents 4.7 or 1.2 fold increase in the diagnostic yield when compared to testing *TPMT* or *NUDT15* only, respectively. Consequently, about one in every four patients tested received a result with recommendation to adjust dose. The majority of recommendations were actioned by the prescriber, resulting in a change to patient’s prescription (54.9%).

While a reactive testing approach was adopted, there is potential economic and logistical advantages to a multi-gene, pre-emptive model in which broader testing of many genes is performed in anticipation of future clinical utility (Bielinski et al., 2014; Ji et al., 2016; Weitzel et al., 2017). In our earlier study on the analysis of four drug-gene interactions in over 500 individuals, a multiplexed test revealed an actionable variant in 98% of genotyped subjects (Goh et al., 2017). This is complemented by findings in this study where multi-gene panel testing would present 272 opportunities for genotype guided treatment optimization in this cohort, primarily for medications metabolized by CYP2D6 and CYP2C19. This finding may be underestimated as our analysis focused on assays which are available for testing in our laboratory. As costs of panel-based genotyping become comparable to single-gene assays, it is logical that a pre-emptive multi-gene panel testing would become increasingly cost effective on a large scale (Bielinski et al., 2014).

From the implementation process of *TPMT* and *NUDT15* testing, we could anticipate barriers associated with scaling the implementation of pre-emptive PGx applications. These included the infrastructure limitations to provide CDS, lack of financial support for development and implementation, and resources for maintenance of a complex system that requires continuous update for testing and reporting in the constantly advancing PGx knowledge (Rohrer Vitek et al., 2017). These will require institutional commitment and collaborative efforts of different stakeholders from administrative, laboratory, pharmacy to clinical informatics. While unfamiliarity with PGx is often cited as a barrier to the adoption of PGx in clinical practice (Chan et al., 2017; Mukerjee et al., 2018), we noted a high degree of acceptance with regards to PGx testing among *TPMT* and *NUDT15* test providers that participated in a questionnaire survey. While the participants indicated that the development of guidelines and provision of education will facilitate the implementation of pre-emptive multi-gene panel testing, actual implementation in practice may still be challenging. As PGx testing can inform future therapeutic decision-making, it is important that both providers and patients understand the significance of the results. Educating patients of varying levels of literacy is needed to minimize misinterpretation. There are concerns on the ground that providers may open themselves to potential litigation with pre-emptive panel testing in the event that the recommendation is not used or the outcome is not as expected. Hence, the potential negative consequences that PGx testing may yield need to be carefully weighed.

Our study is limited by the small number of patients. Hence, we can only evaluate the implementation efficiencies in 51 patients with actionable results. This was based on prescription changes within 3 months of genotyping in their EMR but we cannot account for changes that could be due to side effects rather than the result of the genotyping. For the survey, it was not known whether the 14 who responded are representative of the 98 providers invited as they are not required to provide their clinical specialties.

In conclusion, we showed that *TPMT/NUDT15* testing has real-world value in routine clinical care. Our experience laid the groundwork for subsequent PGx implementations, including pre-emptive multi-gene testing. To establish that, the development of sustainable educational strategists and infrastructure to manage and distribute pharmacogenomic information in the EMR is imperative. As we move forward, we will continue to track implementation metrics to optimize workflow.

## Data Availability

The original contributions presented in the study are publicly available. This data can be found here: TPMT*3B: rs1800460 https://www.ncbi.nlm.nih.gov/snp/rs1800460 TPMT*3C: rs1142345 https://www.ncbi.nlm.nih.gov/snp/rs1142345 NUDT15*3 (c.415C>T): rs116855232 https://www.ncbi.nlm.nih.gov/snp/rs11685232.
