# Supplementary material for: TPMT and NUDT15 testing for thiopurine therapy: A major tertiary hospital experience and lessons learned
Source: Front Pharmacol. 2022 Sep 23;13:837164. doi: 10.3389/fphar.2022.837164 (PMC9537458; doi:10.3389/fphar.2022.837164)
Supplement: Supplementary file 1 [file Table1.DOCX]

**Supplementary Table 1.** List of medications with actionable PGx guidance and available for gene testing.

| **Drug** | **Gene** |
| --- | --- |
| Azathiopurine | NUDT15 TPMT |
| Abacavir | HLA-B *57:01 |
| Allopurinol | HLA-B *58:01 |
| Amitriptyline | CYP2C19 CYP2D6 |
| Carbamazepine | HLA-A *31:01 HLA-B *15:02 |
| Clopidogrel | CYP2C19 |
| Codeine | CYP2D6 |
| Escitalopram | CYP2C19 |
| Fluvoxamine | CYP2D6 |
| Mercaptopurine | NUDT15 TPMT |
| Nortriptyline | CYP2D6 |
| Ondansetron | CYP2D6 |
| Phenytoin | CYP2C9 HLA-B *15:02 |
| Simvastatin | SLCO1B1 |
| Tacrolimus | CYP3A5 |
| Tramadol | CYP2D6 |
| Thioguanine | NUDT15 TPMT |

CYP, cytochrome; HLA, Human leukocyte antigen; SLCO1B1, solute carrier organic anion transporter family member 1B1
