# Supplementary figures and images for: TPMT and NUDT15 testing for thiopurine therapy: A major tertiary hospital experience and lessons learned
Source: Front Pharmacol. 2022 Sep 23;13:837164. doi: 10.3389/fphar.2022.837164 (PMC9537458; doi:10.3389/fphar.2022.837164)

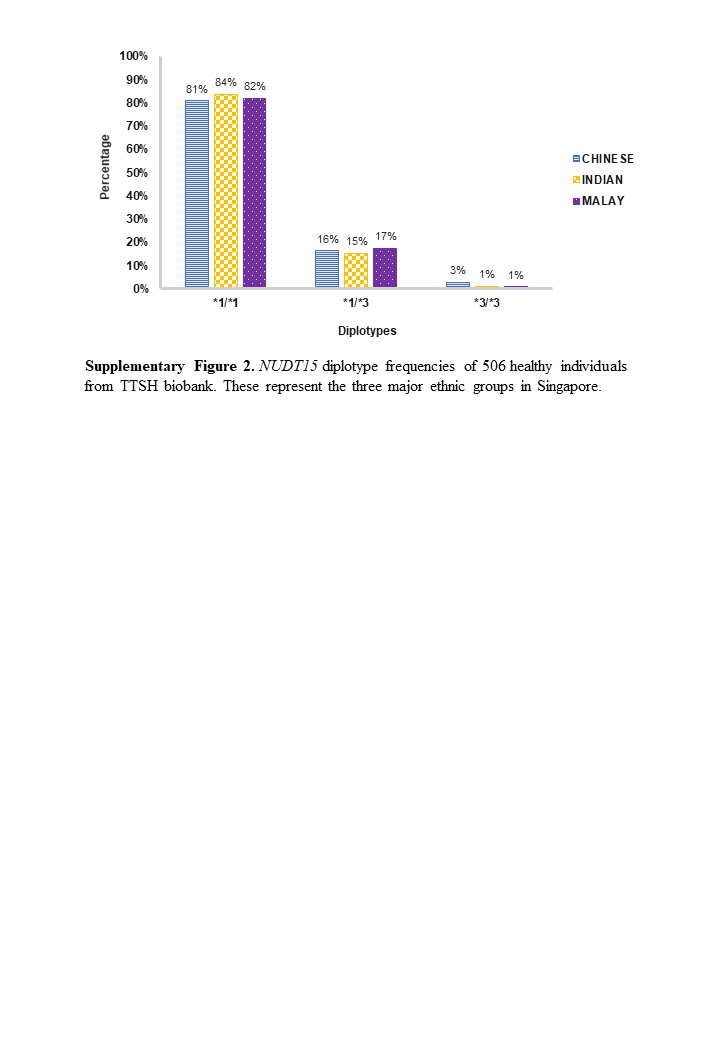

Supplement: Supplementary file 2 [file Image2.TIF]

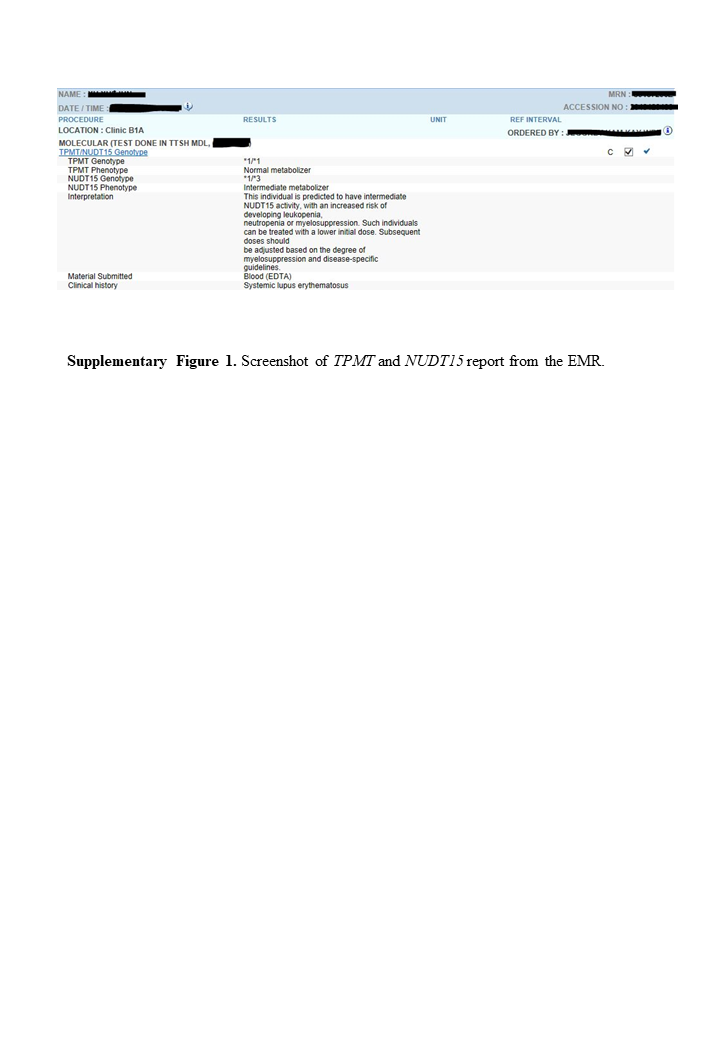

Supplement: Supplementary file 3 [file Image1.TIF]
